# Factors Associated With Receipt of Smoking Cessation Advice and Assistance by Health Professionals Among Latino and Non-Latino White Smokers With Medicaid Insurance in California

**DOI:** 10.1001/jamanetworkopen.2021.44207

**Published:** 2022-01-19

**Authors:** Cindy V. Valencia, Melanie Dove, Elisa K. Tong

**Affiliations:** 1 Department of Internal Medicine, University of California, Davis, Sacramento; 2 Department of Public Health Sciences, University of California, Davis, Davis

## Abstract

**Importance:**

Nationally, Latino smokers are less likely than non-Latino White smokers to receive advice and assistance from health professionals to quit smoking. California’s Medicaid expansion included the Patient Protection and Affordable Care Act’s comprehensive tobacco cessation benefits; however, it is unknown whether expanded coverage helped resolve this disparity.

**Objective:**

To examine the association between race and ethnicity (Latino and non-Latino White) and health professional cessation advice and assistance among smokers with Medi-Cal insurance in the post–Affordable Care Act period.

**Design, Setting, and Participants:**

This repeated cross-sectional study was conducted with the 2014 and 2016-2018 California Health Interview Survey. A total of 1861 Latino and non-Latino White current smokers aged 18 to 64 years who had Medi-Cal insurance and consulted a health professional in the past 12 months were included. Data were analyzed between December 1, 2019, and April 30, 2021.

**Exposure:**

Race and ethnicity classified as Latino or non-Latino White.

**Main Outcomes and Measures:**

The outcomes were receipt of health professional advice to quit smoking or assistance to quit in the past 12 months. Logistic regression was used to examine the association between race and ethnicity and each outcome, adjusted for sociodemographic factors, smoking behavior, health care factors, and acculturation measures. All estimates were weighted to adjust for the complex survey design.

**Results:**

Among 1861 participants, 44.8% were Latino, 53.8% were aged 40 years or older (mean [SE], 39.7 [0.79] years), 54.1% were male, and 59.9% had less than a high school education. Latino smokers were less likely than non-Latino White smokers to receive health professional advice (38.3% Latino smokers vs 55.3% non-Latino White smokers) or assistance (21.8% Latino smokers vs 35.7% non-Latino White smokers). In the unadjusted model, compared with non-Latino White smokers, Latino smokers were less likely to receive advice (odds ratio [OR], 0.50; 95% CI, 0.29-0.86) and also less likely to receive assistance (OR, 0.50; 95% CI, 0.25-1.00). However, in the adjusted model, race was no longer significant. Smokers with more office visits (adjusted OR, 2.44; 95% CI, 1.61-3.70) and those with at least 1 chronic disease (adjusted OR, 1.99; 95% CI, 1.15-3.43) were more likely to receive advice from a health professional. Additionally, daily smokers compared with nondaily smokers (adjusted OR, 2.29; 95% CI, 1.03-5.13) were more likely to receive assistance.

**Conclusions and Relevance:**

In this cross-sectional study, more office visits, having a chronic disease, and daily smoking were associated with an increased likelihood of receiving smoking cessation advice or assistance. Use of strategies to engage tobacco users outside of the clinic, such as proactive outreach and community-based engagement, may help address this disparity.

## Key Points

**Question:**

What are the underlying factors associated with disparities between California Latino and non-Latino White smokers with Medicaid insurance who receive advice and assistance from health professionals regarding smoking cessation?

**Findings:**

In this cross-sectional study of 1861 patients, Latino smokers were less likely to receive advice from clinicians in the unadjusted model. When adjusted for sociodemographic factors, smoking behavior, health care factors, and acculturation measures, the difference was not statistically significant.

**Meaning:**

Efforts to eliminate disparities for Latino smokers might consider community engagement and population health outreach strategies outside the clinical encounter.

## Teaser

This cross-sectional study uses the California Health Interview Survey to investigate disparities in receipt of smoking cessation advice and assistance between Latino and non-Latino White smokers.

## Introduction

Tobacco use is still a major cause of morbidity and mortality in the United States.^1^ Nationally, 14.0% of adults are cigarette smokers, and people covered by Medicaid insurance have higher smoking rates than the general population.^2^ Although smoking prevalence among Latino adults (8.8%) is lower than among White adults (15.5%),^3^ considering only smoking prevalence misses the high total numbers of smokers in the large Latino population, and prevalence also varies within Latino subgroups.^4^ Latino individuals are the largest and second-fastest-growing racial and ethnic minority group in the United States. A better understanding of Latino tobacco use and cessation behaviors is needed to inform prevention, screening, and treatment strategies.

Access to health insurance coverage and health care services, including tobacco cessation treatment, is a known barrier among Latino individuals,^5^ but through the Patient Protection and Affordable Care Act (ACA), many of them gained health care access. Few studies have examined whether tobacco treatment disparities persist in the post-ACA era, or specifically between Latino and non-Latino White smokers. Pre–Affordable Care Act studies reported that Latino smokers were less likely than non-Latino White smokers to receive advice to quit and cessation assistance from a health professional^6,7,8,9^; these studies examined racial and ethnic differences across the overall population. Only 1 national post-ACA study has explored how these trends compare among Latino and non-Latino White adults, which reinforced findings of previous studies that reported continued disparities between Latino and non-Latino White adults in advice from health professionals to quit.^10^ A regional post-ACA study of newly enrolled Kaiser Permanente patients in Northern California^11^ reported that Spanish-speaking, light/nondaily smokers had significantly lower rates of advice from a health professional to quit (56%) compared with English-speaking patients who were light/nondaily smokers (84%). Similarly, Spanish-speaking daily smokers reported lower rates of advice from a health professional to quit (70%) compared with English-speaking daily smokers (84%). The study findings highlight the importance of assessing language and smoking intensity among Latino populations to provide comprehensive services.

In California, the ACA expansion increased access to Medi-Cal insurance to 2.8 million people by the fall of 2014; of these, 41% were newly insured nonelderly Latino individuals,^12^ and the percentage of smokers covered by Medi-Cal increased substantially, from 19.3% before the ACA to 41.5% afterward.^13^ Therefore, there is a need to assess smoking intensity, access to cessation advice, and acculturation measures to better understand Latino tobacco use and treatment in Medi-Cal. Acculturation describes the process of acquiring cultural elements (eg, behaviors, attitudes, norms, values) of the host culture.^14^ Survey studies show that Latino individuals with a higher level of acculturation (as measured by US birthplace or English-language proficiency) are more likely to be daily smokers and smoke more cigarettes per day than foreign-born Latino individuals.^15,16,17,18^ Latino individuals also have the highest rates of nondaily smoking,^15^ which is associated with being less likely to be advised or assisted by health professionals to quit.^19^

This study compares health professional advice and assistance in the post-ACA period among Latino and non-Latino White smokers with Medi-Cal and investigates associated factors. California provides a unique setting to examine Latino tobacco use, screening, referrals, and cessation behaviors because it has approximately 3 million smokers, one-third of whom are Latino. We hypothesized that race and ethnicity, lower acculturation, and lower smoking intensity (eg, nondaily smoking) may be associated with receiving less health professional advice and cessation assistance.

## Methods

### Design

Data from the 2014 and 2016-2018 California Health Interview Survey (CHIS), a population-based survey that is representative of the California population, were analyzed as a repeated cross-sectional study. Primary outcomes were not included in CHIS 2015, 2019, or 2020, and therefore these years were not included in this secondary analysis. The CHIS is a stratified random-digit-dial telephone survey of landline-only and cell phone–only households, with more than 20 000 participants each year. The survey is conducted in English, Spanish, Chinese (Cantonese and Mandarin), Korean, and Vietnamese. The overall adult survey response rate was 44.8% in 2014, 41.5% in 2016, and 42.3% in 2017 and 2018. The CHIS is certified by the American Association for Public Opinion Research (AAPOR) Transparency Initiative and follows their reporting guidelines as outlined in their methodology report.^20^ This study followed the Strengthening the Reporting of Observational Studies in Epidemiology (STROBE) reporting guideline. The need for informed consent was waived by the University of California, Davis institutional review board because this study was approved as exempt from human subjects review.

### Sample

The study inclusion criteria included adults reporting insurance coverage with Medi-Cal, aged 18 to 64 years (up to the Medicare eligibility cutoff), who self-identified as Latino or non-Latino White, had a clinician visit in the past 12 months, and currently smoked cigarettes (N = 1861). Current smokers were defined as ever having smoked 100 cigarettes in their lifetime and smoked every day or some days in the past 30 days.

### Outcome

To assess advice to quit smoking and assistance from a health professional with referral or cessation information, smokers were asked the following: “In the past 12 months, did a doctor or other health professional advise you to quit smoking?” and “In the past 12 months, did a doctor or other health professional refer you to, or give you information about, a cessation program?” These questions were administered only to current smokers in the survey.

### Covariates

Covariates reflect 4 domains that may influence health professionals’ advice and assistance: sociodemographic factors, smoking behavior, measures of acculturation, and health care factors. Sociodemographic variables included age (18-29 years, 30-39 years, 40-49 years, and 50-64 years), sex (male vs female), education (less than high school education or high school graduate vs any college), and race and ethnicity (Latino and non-Latino White). Smoking behavior variables included smoking intensity (daily or nondaily smoking), stopped smoking in the past year for 1 day or longer (yes vs no), and thinking about quitting in the next 6 months (yes vs no). The measures of acculturation included birthplace (the United States or foreign born) and English-language proficiency (English only, speaking English well or very well, and not speaking English well or at all). The health care factors reclassification of the chronic health conditions, frequency of physician visits, and psychological distress variables were modeled after the variable categories chosen in a previous CHIS Medicaid smoker study.^13^ The health care factor variables included number of office visits to a “medical doctor” in the past 12 months (1-4 vs ≥5), having at least 1 chronic disease (yes vs no), and experiencing psychological distress in the past year (yes vs no). Participants were classified as having at least 1 chronic disease if they were told by a physician that they had 1 or more of these conditions: heart disease, hypertension, diabetes, or current asthma. The 6-item Kessler Psychological Distress Scale was used to classify adults as experiencing psychological distress. Scores on the 6 dimensions were coded and summed to produce a total score of 0 to 24, with a 0 indicating no distress at all and 24 indicating extreme distress. Scores of 13 or greater were considered as indicating severe psychological distress. The survey year variable was included to assess trends in each outcome during the 2014 and 2016-2018 periods. These variables were selected according to previous study findings.^6,7,8,9,11,13,21^

### Statistical Analysis

The prevalence of each covariate was estimated for Latino and non-Latino White smokers, and differences were assessed with χ^2^ tests. In addition, the prevalence of each outcome was estimated for all covariates, and differences were assessed with χ^2^ tests. Statistical significance was set at 2-sided *P* = .05. Unadjusted and adjusted logistic regression was used to examine the association between race and each outcome. Adjusted models included all covariates described earlier. All estimates were weighted to adjust for the complex survey design, using SAS survey procedures such as survey logistic regression. The CHIS uses replicate weights to adjust for both the weighting and the stratum. Data were analyzed between December 1, 2019, and April 30, 2021. All analyses were conducted with SAS version 9.4 (SAS Institute Inc).

## Results

Table 1 displays the characteristics of Latino and non-Latino White smokers with Medi-Cal who reported consulting a health professional in the past year. Among 1861 participants, 44.8% were Latino, 55.2% were non-Latino White, 53.8% were aged 40 years or older (mean [SE], 39.7 [0.79] years), 54.1% were men, 45.9% were women, and 59.9% had less than a high school education. Compared with non-Latino White smokers, Latino smokers had a higher percentage of nondaily smoking, had fewer office visits, had no chronic disease, had limited English proficiency, and were foreign born. There were no significant differences by quit attempt in the past year or thinking about quitting smoking in the next 6 months. Among Latino and non-Latino White adults with Medi-Cal who smoked, 75.1% and 81.5%, respectively, ever had a physician visit in the past year.

**Table 1.** Characteristics of Latino and Non-Latino White Smokers With California Medicaid, California Health Interview Survey, 2014 and 2016-2018^a^

Characteristic	No. (weighted %)			*P* value^b^
	Total	Latino	Non-Latino White	
Overall	1861 (100)	557 (44.8)	1304 (55.2)	.65
Demographic characteristics				
Age, y				
18-29	273 (22.5)	114 (26.2)	159 (19.4)	.18
30-39	320 (23.7)	106 (26.1)	214 (21.7)	
40-49	325 (18.9)	106 (19.9)	219 (18.2)	
50-64	943 (34.9)	231 (27.8)	712 (40.7)	
Sex				
Women	981 (45.9)	250 (40.9)	731 (50.0)	.33
Men	880 (54.1)	307 (59.1)	573 (50.0)	
Education				
≤High school	1045 (59.9)	378 (67.7)	667 (53.6)	.04
Any post–high school education	816 (40.1)	179 (32.3)	637 (46.4)	
Smoking behavior				
Daily	1310 (65.1)	314 (54.2)	996 (74.0)	.002
Stopped smoking 1 d or longer to quit in the past year	1098 (59.4)	355 (62.3)	743 (57.0)	.39
Thinking about quitting smoking in next 6 mo	1358 (73.0)	410 (71.6)	948 (74.0)	.65
Health care				
≥5 Office visits to physician in past year	905 (40.4)	213 (28.1)	692 (50.4)	<.001
≥1 Chronic disease	1049 (49.3)	290 (41.1)	759 (55.9)	.03
Experienced psychological distress^c^	578 (29.3)	143 (22.7)	435 (34.7)	.008
Acculturation				
English-language proficiency^d^	1727 (88.4)	428 (74.9)	1299 (99.4)	<.001
Born in the US	1610 (78.4)	365 (62.6)	1245 (91.3)	<.001

^a^ Unweighted frequencies and weighted percentages.

^b^ *P* value for χ^2^ test of the difference between Latino and non-Latino White.

^c^ Scored at least 13 of 24 on the 6-item Kessler Psychological Distress Scale.

^d^ Self-rated as English only, or speaking English well or very well.

Among adult smokers with Medi-Cal who had a clinician visit in the past year, more than half had not received advice, and almost three-quarters had not received assistance to quit smoking (Table 2). Compared with non-Latino White smokers, a lower percentage of Latino smokers reported having received advice (38.3% Latino smokers vs 55.3% non-Latino White smokers) and assistance (21.8% Latino smokers vs 35.7% non-Latino White smokers) from a health professional. Groups reporting a higher percentage of receiving advice or assistance included those aged 50 to 64 years, daily smokers, those with at least 1 chronic disease, and those who visited a physician 5 or more times in the past year. There was no change in either advice or assistance from health professionals over time.

**Table 2.** Advice or Assistance from Health Professionals Among Latino and Non-Latino White Smokers With California Medicaid, California Health Interview Survey, 2014 and 2016-2018^a^

Variable	Advice, No. (weighted %)	*P* value^b^	Assistance, No. (weighted %)	*P* value^b^
Overall, No. (weighted %)	1861 (47.7)		1861 (29.5)	
Survey year				
2014	191 (42.1)	.60	118 (43.2)	.15
2016	314 (45.1)		147 (23.1)	
2017	278 (51.0)		157 (30.4)	
2018	310 (51.0)		172 (25.4)	
Demographic characteristics				
Race and ethnicity				
Latino	273 (38.3)	.01	138 (21.8)	.054
Non-Latino White	820 (55.3)		456 (35.7)	
Age, y				
18-29	100 (32.7)	.001	57 (23.5)	.08
30-39	157 (43.5)		77 (21.7)	
40-49	188 (43.5)		107 (29.9)	
50-64	648 (62.4)		353 (38.4)	
Sex				
Women	596 (49.6)	.55	345 (32.5)	.32
Men	497 (46.1)		249 (26.8)	
Education				
≤High school	609 (45.3)	.32	313 (26.9)	.26
Any post–high school education	484 (51.2)		281 (33.3)	
Smoking behavior				
Smoking intensity				
Daily	840 (53.7)	.01	463 (35.1)	.01
Nondaily	253 (36.6)		131 (19.0)	
Stopped smoking 1 d or longer to quit in the past year				
Yes	654 (49.5)	.46	362 (30.9)	.51
No	439 (45.1)		232 (27.3)	
Thinking about quitting smoking in the next 6 mo				
Yes	817 (51.1)	.08	443 (30.3)	.64
No	276 (38.4)		151 (27.2)	
Health care				
No. of office visits to physician in the past year				
1-4	468 (36.0)	<.001	248 (23.0)	.02
≥5	625 (64.9)		346 (39.0)	
Chronic diseases^c^				
≥1	713 (60.7)	<.001	381 (36.1)	.03
None	380 (35.0)		213 (23.0)	
Experienced psychological distress^d^				
Yes	376 (53.9)	.20	196 (30.5)	.80
No	713 (45.0)		398 (29.1)	
Acculturation				
English-language proficiency^e^				
Speak only English, very well or well	1027 (48.5)	.46	562 (29.6)	.93
Do not speak English well or at all	66 (41.3)		81 (28.5)	
Born in the US				
Yes	963 (49.9)	.23	530 (29.7)	.90
No	130 (39.8)		32 (28.6)	

^a^ Unweighted frequencies and weighted percentages.

^b^ *P* value for χ^2^ test of association between each covariate and outcome.

^c^ Reported heart disease, hypertension, diabetes, and current asthma.

^d^ Scored at least 13 of 24 on the 6-item Kessler Psychological Distress Scale.

^e^ Self-rated as English only, or speaking English well or very well.

Results from the unadjusted logistic regression analysis revealed that race and ethnicity were significantly associated with both outcomes (Table 3 and Table 4). Compared with non-Latino White smokers, Latino smokers were half as likely to receive advice from a health professional to quit smoking (odds ratio [OR], 0.50; 95% CI, 0.29-0.86) and to receive assistance (OR, 0.50; 95% CI, 0.25-1.00). In the final adjusted model, there was no statistically significant difference between Latino and non-Latino White smokers (Tables 3 and 4). Smokers with at least 1 chronic health condition were twice as likely to receive advice from a health professional to quit smoking (adjusted OR, 1.99; 95% CI, 1.15-3.43). Similarly, smokers who visited a physician 5 times or more in the past year were 2.4 times as likely to receive advice (adjusted OR, 2.44; 95% CI, 1.61-3.70). Compared with nondaily smokers, daily smokers were 2.3 times as likely to report receiving a referral or cessation information (adjusted OR, 2.29; 95% CI, 1.03-5.13), but daily smoking was not associated with assistance from a health professional to quit smoking (OR, 1.83; 95% CI, 0.95-3.53).

**Table 3.** Multivariable Logistic Regression of Factors Associated With Advice from Health Professionals to Quit by Smoker Characteristics, California Health Interview Survey, 2014 and 2016-2018

Variable	Unadjusted, OR (95% CI) (N = 1861)	*P* value	Adjusted, OR (95% CI) (n = 1857)	*P* value
Race				
Latino	0.50 (0.29-0.86)	.01	0.71 (0.37-1.38)	.31
Non-Latino White	1 [Reference]		1 [Reference]	
Smoking intensity				
Daily	NA	NA	1.83 (0.95-3.53)	.07
Nondaily	NA	NA	1 [Reference]	
No. of visits				
1-4	NA	NA	1 [Reference]	<.001
≥5	NA	NA	2.44 (1.61-3.70)	
Chronic disease				
No	NA	NA	1 [Reference]	.01
≥1	NA	NA	1.99 (1.15-3.43)	
English-language proficiency				
Speaks well/very well	NA	NA	0.60 (0.17-2.15)	.44
Does not speak well/at all	NA	NA	1 [Reference]	
Born in the US				
Yes	NA	NA	0.83 (0.27-2.53)	.74
No	NA	NA	1 [Reference]	

Abbreviations: NA, not applicable; OR, odds ratio.

Adjusted model includes year, age, sex, education, smoking intensity, stopped smoking, thinking about quitting, number of office visits, chronic disease, distressed, and the acculturation variables. Year, age, sex, education, stopped smoking 1 day or longer to quit in the past year, thinking about quitting, and distressed were included in the final model and were not significant (data not shown).

**Table 4.** Multivariable Logistic Regression of Factors Associated With Assistance from Health Professionals by Smoker Characteristic, California Health Interview Survey, 2014 and 2016-2018

Variable	Unadjusted, OR (95% CI) (N = 1861)	*P* value	Adjusted, OR (95% CI) (n = 1857)	*P* value
Race				
Latino	0.50 (0.25-1.00)	.051	0.61 (0.27-1.38)	.23
Non-Latino White	1 [Reference]		1 [Reference]	
Smoking intensity				
Daily	NA	NA	2.29 (1.03-5.13)	.04
Nondaily	NA	NA	1 [Reference]	
No. of visits				
1-4	NA	NA	1 [Reference]	.06
≥5	NA	NA	1.71 (0.99-2.96)	
English-language proficiency				
Speaks well/very well	NA	NA	0.70 (0.11-4.62)	.71
Does not speak well/at all	NA	NA	1 [Reference]	
Born in the US				
Yes	NA	NA	1.18 (0.33-4.24)	.80
No	NA	NA	1 [Reference]	

Abbreviations: NA, not applicable; OR, odds ratio.

Adjusted model includes year, age, sex, education, smoking intensity, stopped smoking, thinking about quitting, number of office visits, chronic disease, distressed, and the acculturation variables. Year, age, sex, education, stopped smoking 1 day or longer to quit in the past year, thinking about quitting, chronic disease, and distressed were included in the final model and were not significant (data not shown).

## Discussion

### Summary

In this study, there was no statistically significant difference in the final adjusted model for the likelihood of receiving smoking cessation advice from a health care professional between Latino and non-Latino White smokers enrolled in Medi-Cal. Five or more visits to a physician, having at least 1 chronic disease, and daily smoking were significantly associated with advice and assistance, suggesting that these factors help explain outcome differences among Latino and non-Latino White Medi-Cal beneficiaries. This research contributes to the understanding of the outcome differences between 2 groups having Medi-Cal and who consulted a health professional in the last year.

Despite an increase in health insurance coverage, there was no change in the percentage of smokers with Medi-Cal who received cessation advice or assistance from 2014 to 2018. This finding is consistent with 1 national post-ACA study that compared Latino and non-Latino White smokers’ receiving advice from health professionals to quit, noting there was no change over time (2000-2015).^10^ Although this national study did not account for any of the health care factors in our study, similarly, the findings from our study confirm there were no significant changes in advice or assistance to Latino smokers over time.

Our finding that daily smoking, compared with nondaily smoking, was associated with receiving smoking cessation assistance is consistent with a regional post-ACA study of newly enrolled Kaiser Permanente patients in Northern California^11^ that adjusted for factors similar to those used in our study, including preferred language. They found that Spanish-speaking nondaily smokers had significantly lower rates of advice (56%) compared with English-speaking smokers and Spanish-speaking daily smokers. Some research suggests there could be a patient-level misconception that infrequent nondaily tobacco use does not qualify one as a tobacco user and may not prompt a physician to provide advice.^22^ However, nondaily, light use could present a long-term elevated risk of tobacco-related illness. A meta-analysis^23^ found that no safe level exists for cardiovascular disease and that even 1 cigarette per day carries risk. Health care systems can implement screening protocols that ask about use in the past 30 days to capture nondaily smokers.

We found that visiting a physician more often was associated with an increase in receiving cessation advice and assistance. In the study, Latino individuals who smoked had slightly lower rates than non-Latino White smokers for consulting a health professional in the past year, and, among those who did so, non-Latino White individuals who smoked were nearly twice as likely to have at least 5 office visits as Latino smokers were. For populations who are less likely to visit a physician, such as Latino individuals, Medicaid plans and health care systems might consider proactive outreach strategies^24^ to help direct individuals to evidence-based state quit-line services. Less than one-fifth of Latino individuals (19.5%) are enrolled in quit-line services, yet they make up more than one-third (34.2%) of all smokers in California.^25^

Use of communication messages and channels that consider the Latino community and culture can help address Latino smoking behavior. Community-based messaging strategies that focus on how secondhand smoke harms the family are motivating messages. Messages should address smoking risk, racial and ethnic differences, and smoke-free homes, and address situational triggers through brief interventions.^19^ Leveraging community leaders and *promotoras* (lay health workers) can be an effective channel for conveying health messages.^26^

Broader and simultaneous strategies to promote cessation services outside of the clinical encounter, which include written materials (eg, in-home mailings) with information about nicotine replacement therapy, are proven strategies and necessary to address higher smoking rates among California’s Medi-Cal population.^27^ Spanish-speaking Latinx individuals had a higher engagement with the California Smokers’ Helpline through in-home mailings (Spanish-speaking Latinx vs White individuals, 30.6% vs 18.2%, respectively).^28^ Once engaged with the helpline, Spanish-speaking Latinx individuals had higher rates of completing counseling and receiving nicotine therapy than White individuals. Targeted Spanish language–designed media can increase calls to the helpline and improve quit outcomes. Greater awareness and research are necessary to improve tobacco cessation advice and assistance in younger, healthier patients and nondaily smokers.

### Limitations

The study has several limitations. Survey response was below 50%, which may lead to nonresponse bias. To help address this bias, CHIS survey weights include an adjustment for nonresponse. The repeated cross-sectional study design precludes causal inferences. There may be misclassification owing to self-report of smoking intensity, which is likely nondeferential. Although the data rely on smokers’ self-reports, which were not biochemically verified with cotinine levels, a meta-analysis comparing self-reported smoking status with results of biochemical validation suggested generally high levels of sensitivity and specificity for self-report.^29^ Additionally, these responses regarding clinician visits may have been influenced by recall bias and potential misclassification also owing to self-report. This study did not examine cessation outcomes, but advice and assistance from health professionals are important process measures toward quitting. Latino tobacco use and cessation behaviors in California may vary from that of other Latino subgroups and Latino individuals in other geographic areas in the United States; therefore, generalizability may be limited. The use of ORs in our analysis may have overestimated the strength of the association when the outcome of interest was not rare (>10%).^30^

## Conclusions

To our knowledge, this is the first study to examine factors associated with Latino vs non-Latino White smokers receiving Medicaid and reporting less advice and assistance from health professionals in the post-ACA era. More office visits, having a chronic disease, and daily smoking were associated with an increased likelihood of receiving smoking cessation advice or assistance. Use of strategies to engage tobacco users outside of the clinic, such as proactive outreach and community-based engagement, may help address this disparity. Future Latino tobacco interventions and population health strategies need to understand the unique smoking behaviors and health care use practices of low-income Latino individuals. The findings may be useful in promoting awareness of these patient-level differences to health care and in informing the design of more targeted studies that can examine the health care system–, clinician-, and patient-level factors in more detail.
